# Investigation of foaming causes in three mesophilic food waste digesters: reactor performance and microbial analysis

**DOI:** 10.1038/s41598-017-14258-3

**Published:** 2017-10-20

**Authors:** Qin He, Lei Li, Xiaofei Zhao, Li Qu, Di Wu, Xuya Peng

**Affiliations:** Key Laboratory of Three Gorges Reservoir Region’s Eco-Environment, Ministry of Education, Chongqing University, Chongqing, 400045 PR China

## Abstract

Foaming negatively affects anaerobic digestion of food waste (FW). To identify the causes of foaming, reactor performance and microbial community dynamics were investigated in three mesophilic digesters treating FW. The digesters were operated under different modes, and foaming was induced with several methods. Proliferation of specific bacteria and accumulation of surface active materials may be the main causes of foaming. Volatile fatty acids (VFAs) and total ammonia nitrogen (TAN) accumulated in these reactors before foaming, which may have contributed to foam formation by decreasing the surface tension of sludge and increasing foam stability. The relative abundance of acid-producing bacteria (*Petrimonas*, *Fastidiosipila*, etc.) and ammonia producers (*Proteiniphilum*, *Gelria*, *Aminobacterium*, etc.) significantly increased after foaming, which explained the rapid accumulation of VFAs and NH_4_^+^ after foaming. In addition, the proportions of microbial genera known to contribute to foam formation and stabilization significantly increased in foaming samples, including bacteria containing mycolic acid in cell walls (*Actinomyces*, *Corynebacterium*, etc.) and those capable of producing biosurfactants (*Corynebacterium*, *Lactobacillus*, *060F05-B-SD-P93*, etc.). These findings improve the understanding of foaming mechanisms in FW digesters and provide a theoretical basis for further research on effective suppression and early warning of foaming.

## Introduction

Anaerobic digestion is the most feasible method for treating food waste (FW) and recovering renewable energy due to its high organic and moisture contents and low calorific value compared with traditional treatments such as incineration or landfills^1^. However, foaming in anaerobic digesters is a major problem that disrupts the stable operation of biogas plants^2–6^. Kougias *et al*.^7^ surveyed 80% of all centralized biogas plants in Denmark (i.e., 16 full-scale biogas plants) and reported that foaming incidents occur up to three times per year in most plants and greatly interfere with normal operations. Moeller *et al*.^8^ and Subramanian *et al*.^9^ reported a similar phenomenon in Germany and America, respectively. Excessive foam may cause operational problems such as pump clogging and digester breakage, environmental problems due to overflowing sludge, and even financial losses due to reduced biogas production and extra maintenance cost^7–9^. Thus, studies on the causes of foaming in anaerobic digesters are crucial to provide a theoretical basis for further research on effective foaming suppression and prevention measures.

Many studies have suggested that unsuitable or unstable operating conditions such as organic overloading, temperature fluctuation, and inadequate mixing contribute to anaerobic digestion foaming^7–11^. Other studies have indicated that excess surface active materials such as extracellular polymeric substances (EPS), lipids, proteins, and volatile fatty acids (VFAs) decrease surface tension and enhance foaming potential^10,12^. However, as in all biological reactors, microorganisms play a key role in anaerobic digesters^13,14^. Therefore, researchers have begun to analyze the effects of key microorganisms to identify the causes of foaming. Many common filamentous bacteria found in activated sludge are induced by the use of sewage sludge as inoculum or substrate. For example, *Gordonia* and *Microthrix sp*. have been found to proliferate after foaming events and largely contribute to foaming^2,9–11^. However, the cause of foaming in anaerobic digestion systems that use substances other than sewage sludge as substrate is unclear. Kougias *et al*.^15^ investigated the bacterial community in a thermophilic manure-based biogas reactor after a foaming event induced by excess organic loading. They reported that in addition to the well-known foaming bacteria *Nocardia* and *Desulfotomaculum*, the relative abundance of several non-filamentous *Lactobacillus* and *Bacillus* spp., which produce biosurfactants, and *Micrococcus* and *Streptococcus*, which decrease media surface tension, increased significantly after foaming. However, because microbial communities are sensitive to operational conditions, the microbial community structure in foaming digesters with different temperatures or substrates may vary from those found in the studies mentioned above^13,15^. Foaming is also prone to occur in anaerobic digesters used to treat FW^5,6^. As far as we know, most studies have reported only the occurrence of foaming in such systems, whereas few have investigated the changes in microbial communities before and after foaming events or specific microorganisms that cause foaming.

**Figure 1:**
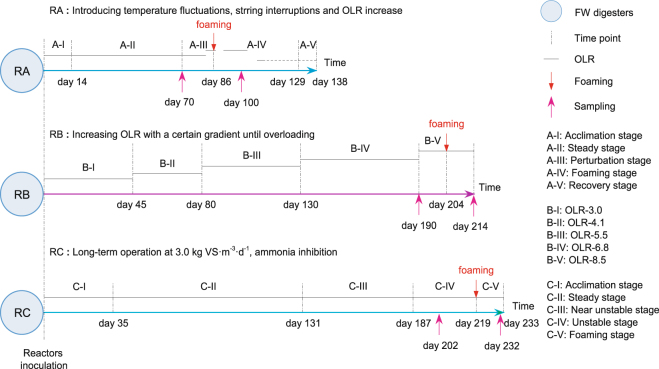
Schematic diagram of the experimental operation. Organic loading rate (OLR) over time in three reactors (RA, RB, and RC) treating food waste (FW) under mesophilic temperature (37 °C ± 1 °C); OLR: organic loading rate (black solid lines) in kg VS·m^−3^·d^−1^. A-I to A-V, B-I to B-V, and C-I to C-V represent operation stages for RA, RB, and RC, respectively. Samples before and after foaming were chosen for microbial community analysis; sampling during stages A-III and A-IV for RA, stages B-IV and B-V for RB, and stages C-IV and C-V for RC.

Due to differences in digester conditions as mentioned above, we could not directly use existing research results to analyze the causes of foaming in mesophilic anaerobic digesters used to treat FW. To identify the universal causes of foaming in mesophilic FW digesters, FW fermentation was carried out through wet digestion in a completely stirring tank reactor (CSTR) (RA) and through dry digestion in a plug flow reactor (PFR) (RB) and CSTR (RC). Foaming was induced in these digesters by temperature fluctuation and mixing interruption (RA), organic overloading (RB), and ammonia inhibition (RC) (Fig. 1). To elucidate the factors contributing to foam formation in these three reactors, physicochemical indicators were monitored during fermentation and microbial community structures before and after foaming were investigated using MiSeq high-throughput sequencing technology. This study provides a theoretical basis for timely foam suppression and effective early warning in anaerobic digesters used to treat FW.

## Results and Discussion

### Reactor performance

**Figure 2:**
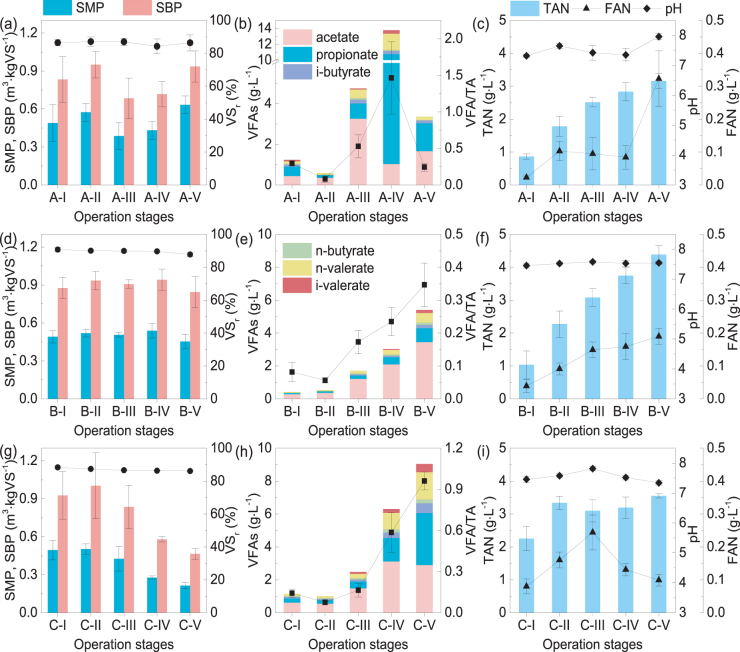
Performance parameters for RA (**a**–**c**), RB (**d**–**f**), and RC (**g**–**i**). Digestion efficiency indicators including SMP, SBP, and VS_r_ (**a**,**d**,**g**), and stability indicators including VFA/TA, VFAs (**b**,**e**,**h**), TAN, FAN, and pH (**c**,**f**,**i**) are presented. A-I to A-V, B-I to B-V, and C-I to C-V respectively represent operation stages for RA, RB, and RC as shown in Fig. 1. SMP: specific methane production; SBP: specific biogas production; VS_r_: volatile solid reduction; VFAs: volatile fatty acids; VFA/TA: the VFA to total alkalinity ratio; TAN: total ammonia nitrogen; FAN: free ammonia nitrogen.

Operation conditions for the three FW digesters are shown in Fig. 1. The digesters were initiated at the same time and acclimated to the same organic loading rate (OLR) of 3.0 kgVS·m^−3^·d^−1^. Different disturbance conditions were used to induce foaming. Temperature fluctuation, mixing interruption, and increased OLR were introduced into RA; the OLR in RB was increased with a specific gradient until organic overloading was induced; RC was operated under the initial OLR and experienced ammonia accumulation and inhibition. Efficiency and stability parameters for each reactor were monitored during operation (Fig. 2).

RA operated stably at 3.0 kgVS·m^−3^·d^−1^ during the first 70 days (acclimation and stable stages) (Fig. 2a). Temperature fluctuations (sludge temperature decrease from 37 °C ± 1 °C to 30 °C ± 1 °C) and mixing interruptions were introduced in RA for a 10 h duration on days 71, 72, and 75, and little foam appeared on the reactor surface. The OLR was increased to 4 gVS·L^−1^·d^−1^ on day 83. Intense foam appeared on day 87 and lasted for 43 days. During the perturbation stage, VFAs rapidly accumulated to 4.65 ± 1.52 (s.d.) g·L^−1^, which was significantly higher than the concentration during the steady stage (p = 0.00) (Fig. 2b). During the foaming stage, VFAs continually accumulated up to 13.45 ± 4.08 (s.d.) g·L^−1^ with a maximum concentration of 18.78 g·L^−1^. The total VFA concentration in the foam layer was much higher than that in the liquid layer during the foaming phase (e.g., 14.28 g·L^−1^ in the upper foam layer and 12.36 g·L^−1^ in the lower liquid layer on day 95). VFAs with more than two carbons accounted for 90.51% ± 8.61% of total VFAs, with propionate accounting for 67.21% ± 11.84%, indicating that propionic acid substituted for acetate as the dominant VFA (Fig. 2b). We hypothesized that VFA accumulation contributed to foam formation in this bioreactor. VFAs have structures with free polic ends (carboxylic ends) that exhibit surfactant properties; thus, they tend to rise with fine gas bubbles and accumulate at the air–liquid interface. Accumulated VFAs could decrease the surface tension of digested liquid, increase foaming tendency, and thereby may promote foam formation^10,12^. The change in VFA composition may have been due to accumulation of electrons by acidogenic bacteria using VFAs with more carbons (e.g., propionate or n-valerate) after H_2_ accumulation in the system^16^. Similarly, accumulation of VFAs was also observed before or during foaming in RB and RC, and VFAs with more carbons such as propionate obviously increased (Fig. 2e,f). This phenomenon has also been reported in other studies. Zhang *et al*.^6^ observed a similar shift in dominant VFAs in a FW digester, and a severe foaming incident occurred after rapid VFA accumulation (to 20.00 g·L^−1^). Ortner *et al*.^4^ reported VFA accumulation prior to foaming in a slaughterhouse waste digester, and VFA concentration maintained a high level of 12.50 g·L^−1^ during foaming.

In addition, total ammonia nitrogen (TAN) in the three reactors exhibited sustained accumulation (Fig. 2c,f,i). Many studies have shown that ammonia accumulation can inhibit process stability, especially the cytotoxic free ammonia nitrogen (FAN), causing cytoplasmic K^+^ loss via an ammonia/K^+^ exchange reaction^17,18^. However, methanogens are sensitive to ammonia inhibition, especially acetate-consuming methanogens. Therefore, ammonia inhibition is often manifested as VFA accumulation. As mentioned above, VFA accumulation promotes foam formation. In the present study, the gradual accumulation of FAN led to rapid VFA accumulation in RC. The VFA build-up reduced the pH to 7.52 ± 0.08 (s.d.), which shifted the TAN equilibrium from predominance of free ammonia to the less inhibitory dissociated form (ammonium ion), leading to a decrease in the inhibition of methanogenic activity (Fig. 2h,i). However, VFAs continued to accumulate, and dense foam formed in this reactor during days 220–233. Lv *et al*.^3^ also observed TAN accumulation (up to 9.56 g·L^−1^) followed by VFA accumulation (up to 36.20 g·L^−1^). In addition, accumulated NH_4_^+^ may also be associated with foam formation. NH_4_^+^ can form ammonium soaps with long-chain fatty acids (i.e., ionic compounds with both hydrophobic and hydrophilic ends in solution) and act as surface active material to promote the formation of foam. Other researchers have also reported that ammonia nitrogen contributes to foam formation. Boe *et al*.^12^ reported that NH_4_^+^ increases foam formation and stability. Moeller *et al*.^8^ found that high ammonia content in feedstock is a main cause of foaming in biogas plants in Germany.

### Microbial community

To elucidate the changes in microbial community structure before and after foaming, the archaeal and bacterial community compositions in each reactor were characterized using Illumina MiSeq high-throughput sequencing. As listed in the Supplementary Table S1, Good’s coverage was greater than 0.99 in both bacterial and archaeal communities, suggesting that the sequencing depth could reveal most bacterial and archaeal communities in the samples.

**Figure 3:**
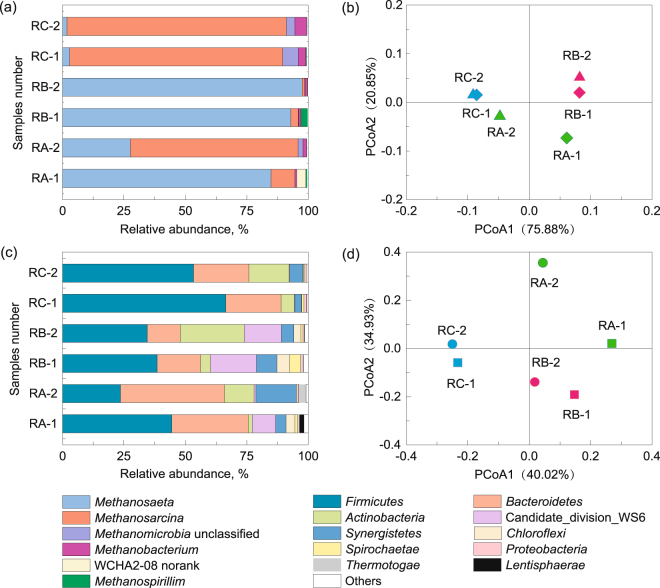
Taxonomic compositions (**a,c**) and principal coordinates analysis (PCoA) (**b,d**) of archaeal and bacterial communities in three digesters. Relative abundance is defined as the number of sequences affiliated with that taxon divided by the total number of sequences per sample (%). Only bacterial phyla or archaeal genera with a proportion higher than 1% in at least one sample are illustrated.

Different methanogens were predominant in the reactors before and after foaming (Fig. 3a). RA and RB were both predominated by the acetoclastic methanogen *Methanosaeta*, whereas the mixotrophic methanogen *Methanosarcina* was the most abundant archaeal genus before foaming in RC. The predominant methanogens in RA shifted to *Methanosarcina* after foaming, but *Methanosaeta* and *Methanosarcina* remained dominant in RB and RC, respectively. Differences in predominant methanogens may be due to variation in reactor configuration or inhibitory substances among the three reactors. *Methanosarcina* was predominant in RA after foaming with high VFA and ammonia accumulation, due to its strong tolerance to high ammonia and VFA concentrations compared with *Methanosaeta* ^17,19,20^. Because the plug flow operation mode of RB protected multicellular aggregates formed by *Methanosaeta* from shear and turbulence and these aggregates could be used to resist inhibition caused by VFA and TAN, *Methanosaeta* was able to maintain high relative abundance during the foaming stage^19^. The predominance of *Methanosarcina* in RC both before and after foaming was probably due to the accumulation of TAN during the early stages of digestion, which supported proliferation of *Methanosarcina* before foaming. According to principal coordinate analysis (PCoA), no obvious changes in the archaeal communities of samples from RB or RC were found before and after foaming (Fig. 3b). Therefore, it is not possible to relate methanogens to foam formation, which supports the results of Kougias *et al*.^15^.

In contrast, bacteria played a major role in the formation of foam^10,15^. *Firmicutes* and *Bacteroidetes* were the most common major phyla in the three reactors before foaming, accounting for 56.2% to 89.0% of total sequences (Fig. 3c). Members of these two phyla are closely related to hydrolysis and acidification of organic substrates and they have been reported as predominant phyla in other mesophilic anaerobic digesters treating FW^13,19,21^. *Firmicutes* decreased in relative abundance in all three reactors after foaming, especially in RA. The proportion of another major phylum *Bacteroidetes* did not obviously change in RC, but substantially increased and decreased in RA and RB, respectively. Moreover, the relative abundance of *Actinobacteria* obviously increased in the three reactors after foaming, reaching up to 12.17%, 26.03%, and 16.39% in RA, RB, and RC, respectively. Most members of *Actinobacteria* have a filamentous structure, which aides in the hydrolysis and acidification processes of anaerobic digestion, and they also are able to metabolize many kinds of substrates, including protein, lipid, lignin, cellulose, sugar, and amino acid^22,23^. The lower abundance of *Actinobacteria* in RA and RC compared with RB may be due to the negative effect of shear and turbulence on the growth of filaments in these two CSTRs. The relative abundance of *Synergistetes* increased by 295% and 106% after foaming in RA and RC, respectively. Its members mainly include anaerobic amino-acid-degrading bacteria and syntrophic acetate-oxidizing bacteria that can be co-cultured with methanogens^24^. Moreover, evident changes in microbial communities were observed after foaming (Fig. 3d). To evaluate the correlations between these changes and foaming as well as identify specific microorganisms related to foam formation, we further analyzed the changes in bacterial genera after foaming.

### Bacterial analysis at the genus level

**Figure 4:**
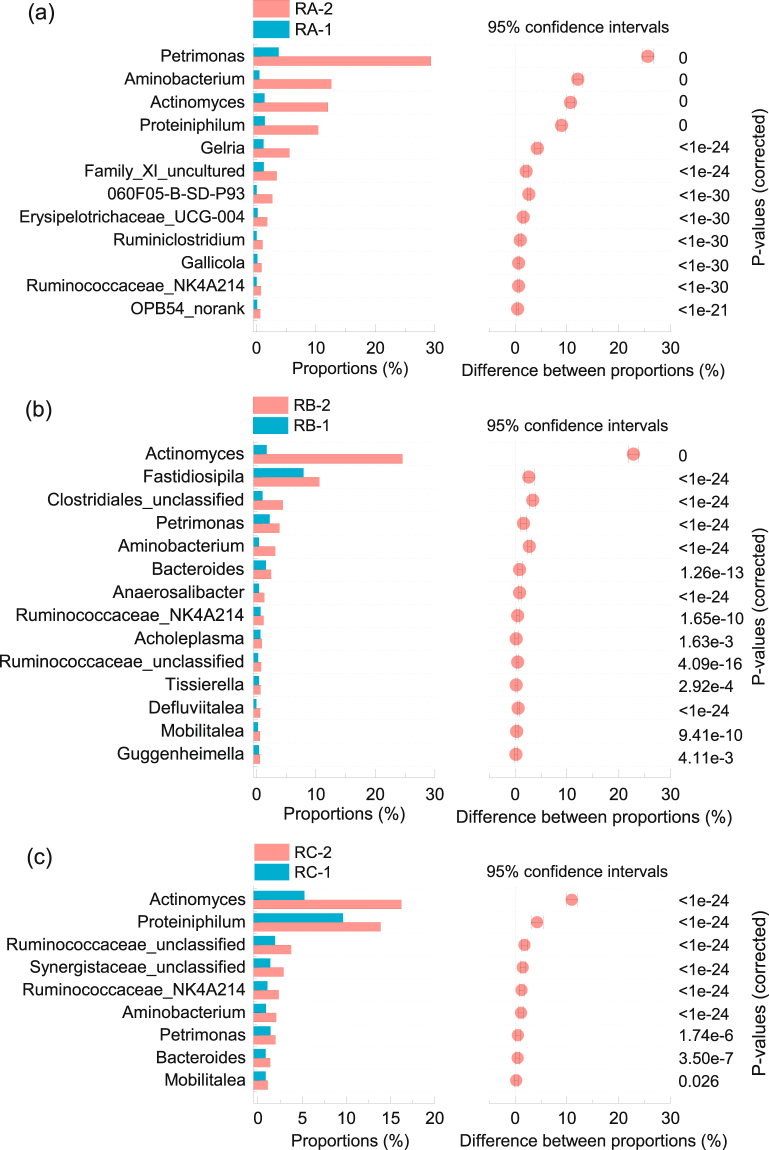
Relative abundance and statistical analysis of the difference between relative abundances of bacterial communities in RA (**a**), RB (**b**), and RC (**c**) before and after foaming. Only bacterial genera that significantly increased in abundance after foaming and with relative abundance higher than 0.5% in foaming samples are represented.

The relative abundances of many genera obviously increased after foaming (Supplementary Tables S2–S4; Fig. 4). In RA, the relative abundance of *060F05-B-SD-P93* was 180 times higher after foaming than before (Fig. 4a). This genus can produce extracellular polymeric substances (EPS), which promote the formation of stable cellular aggregates and facilitate interspecies hydrogen transfer^14^. EPS consists of organic macromolecules produced extracellularly by microorganisms and has been found in the intracellular space of microbial aggregates. The main components of EPS such as polysaccharides and proteins can form ionisable compounds with hydrophobic and hydrophilic ends in solution. Therefore, EPS tends to form an organic film at the air–liquid interface, with the hydrophobic end toward the gas phase and the hydrophilic end toward the liquid phase. This organic film reduces the surface tension of the air–liquid interface and increases the stability of foam^10,12^. Collivignarelli *et al*.^25^ reported that foam formation in a thermophilic membrane reactor was correlated to the presence of EPS, in particular the soluble protein fraction, which was consistent with the results of Cosenza *et al*.^26^ and Di Bella *et al*.^27^. In this study, the relative abundance of *Actinomyces* increased by 802% after foaming. Similar to *Gordonia*, *Microthrix*, and other common foam-forming bacteria in activated sludge systems, *Actinomyces* strains found in the three reactors included filamentous bacteria; the presence of mycolic acids on the cell walls of these bacteria made cells extremely hydrophobic^9,10,28,29^. Due to their hydrophobic properties and potential for biosurfactant production, filaments tend to attach to biogas bubbles and accumulate on the air–liquid interface, resulting in lower surface tension of sludge and enhanced foaming^15,30^. Therefore, proliferation of *Actinomyces* was correlated to the formation of foam.

In addition, many acid-producing bacteria significantly proliferated after foaming in RA (Fig. 4a). The typical cellulose-degrading bacteria *Ruminiclostridium* increased by 7002%. These bacteria can degrade cellulose or hemicellulose polysaccharides to produce acetate and lactate due to its ability to synthesize large extracellular multi-enzyme complexes termed cellulosomes^31^. The relative abundance of *Ruminococcaceae*_NK4A214, a novel rumen bacterium within the family *Ruminococcaceae*, increased by 3926%^32^. *Caldicoprobacter*, which is able to ferment various saccharides to produce lactate and acetate, increased by 878% (Supplementary Table S2)^33^. The relative abundance of *Petrimonas* was highest (29.34%) in foaming samples, and this value was 689% higher than that before foaming. Members of this genus can hydrolyze many types of carbohydrates and organic acids in the presence of elemental sulfur to produce a large amount of acetic acid^34^.

High proliferation of proteolytic bacteria was observed in foaming samples from RA. *Aminobacterium*, which increased by 2583% after foaming, is a typical amino acid-degrading anaerobic bacterium that can ferment various amino acids (alanine, valine, leucine, etc.) when co-cultured with hydrogen-utilizing methanogens^35^. Similarly, the glutamate-degrading bacterium *Gelria* increased by 371% after foaming and can degrade a variety of amino acids and sugars in co-culture with hydrogenotrophic methanogens to yield NH_4_^+^, H_2_, and propionic acid as the main products^36^. The obligate anaerobic proteolytic bacterium *Proteiniphilum* increased in proportion by 619%. Members of this genus can ferment yeast extract, peptone, pyruvate, and L-arginine, yielding NH_3_ and acetic acid as the main products^34^. The relative abundance of *Gallicola*, which is able to metabolize peptone and amino acids to NH_3_, acetic acid, and butyric acid, increased by 456%^37^.

In addition to proliferation of *Actinomyces* after foaming in RB (Fig. 4b), the proportion of *Corynebacterium*, which belongs to the phylum *Actinobacteria*, increased by 55% (Supplementary Table S3). *Corynebacterium* is a genus within the family *Corynebacteriaceae*, classified to the order *Corynebacteriales*, which includes widely known foaming bacteria such as *Gordoniaceae*, *Nocardiaceae*, and *Mycobacteriaceae*. Members of the order *Corynebacteriales* typically contain mycolic acids in their cell walls, and *Corynebacterium* was no exception, containing short-chain mycolic acids with a length of 22–36 carbon atoms (corynemycolic acids)^29^. *Corynebacterium* can also produce biosurfactant-like phospholipids^29^. In addition, *Petrimonas*, *Ruminococcaceae*_NK4A214, *Aminobacterium*, and *Caldicoprobacter*, which significantly increased in RA, respectively increased by 75%, 77%, 696%, and 318% in RB after foaming (Fig. 4b, Supplementary Table S3). Acid-producing bacteria, such as *Defluviitalea*, *Anaerosalibacter*, *Mobilitalea*, *Fastidiosipila*, *Guggenheimella*, and *Bacteroides* also remarkably increased in RB after foaming (Fig. 4b, Supplementary Table S3)^37–41^. The relative abundances of *Tissierella* and *Tepidimicrobium*, which can metabolize proteins and amino acids, increased by 54% and 441% after foaming, respectively^37,42^.

*Lactobacillus*, which can ferment various carbohydrates to produce lactic acid as the main fermentation product, increased by 1151% after foaming in RC (Supplementary Table S4)^37^. Lactic acid is widely used as a foaming agent in the food industry^15^. In addition, *Lactobacillus* spp. also produces biosurfactants during fermentation (mainly proteinaceous, glycolipidic, glycoproteins, or glycolipopeptides)^43,44^. Kougias *et al*. also observed proliferation of this genus during foaming in thermophilic reactors used to treat manure^15^. *Ruminococcus*, which can secrete extracellular enzymes for degradation of cellulose or hemicellulose to produce formic and acetic acid, increased in proportion by 1029% in RC after foaming (Supplementary Table S4)^45^. *Sphaerochaeta* increased by 110% after foaming, and this genus can ferment carbohydrates to produce acetate, formate, and ethanol^46^. Finally, *Actinomyces*, acid-producing bacteria (*Mobilitalea*, *Petrimonas*), and ammonia-producing bacteria (*Proteiniphilum*, *Aminobacterium*, *Bacteroides*, and *Ruminococcaceae*_NK4A214) proliferated in RC after foaming, similar to the trends in RA and RB (Fig. 4c).

In summary, intense foam was induced in the three FW digesters by different methods including temperature disturbance/mixing interruption, organic overloading, and ammonia inhibition. Proliferation of *Actinomyces*, which can contain mycolic acids on its cell walls, was observed after foaming in all three reactors. Bacterial genera capable of producing surface active materials proliferated after foaming in these three reactors. For example, EPS-producing *060F05-B-SD-P93* increased in RA; *Corynebacterium*, which is capable of producing biosurfactant (phospholipids) and containing mycolic acid on its cell walls proliferated in RB; and *Lactobacillus*, which can synthesize foaming agents (lactate) and large amounts of biosurfactants, increased in RC. Extremely hydrophobic filaments or their products with surface active properties increased foam formation and stability. In addition, the relative abundance of acid-producing bacteria (*Petrimonas*, *Fastidiosipila*, etc.) and ammonia producers (*Proteiniphilum*, *Gelria*, *Aminobacterium*, etc.) increased after foaming, which may help to explaine the rapid accumulation of VFAs and NH_4_^+^ after foaming. As shown previously, high concentrations of VFAs may greatly reduce the surface tension of liquid and increase foaming tendency. NH_4_^+^ may promote foam formation and stability as well as inhibit VFA consumption, thereby promoting VFAs accumulation and further strengthening the foaming potential. Therefore, the proliferation of acids and ammonia producers may potentially contribute to foam formation in reactors. The association between foaming and these specific microorganisms requires to be further investigated. Further works is needed to investigate the corresponding foaming thresholds of these specific bacteria (e.g., *Actinomyces*) and evaluate the feasibility of these bacteria as early warning indicators. This information can help realize effective early warning and control methods for foaming during anaerobic digestion of FW.

### Measures for foam prevention and suppression

This survey of foaming in FW digesters does not provide definitive proof of universal foaming agents due to lack of replication by reactor type. But it can be useful for developing hypotheses that foaming in the mesophilic digesters were likely involved a combination of accumulated surface active materials (such as VFAs and NH_4_^+^) and proliferation of specific microorganisms. And further works with more rigorous experimental designs is needed to verify the hypotheses raised in this study. Based on the hypotheses, we present recommendations for foam prevention and suppression below.

Fluctuations in operational conditions (e.g., mixing disturbance and temperature fluctuation), excessive organic loading, and accumulation of inhibitors, which may cause fluctuations in methanogenic activity, must be prevented to avoid VFA accumulation. And the prevention of ammonia inhibition can be achieved by adjusting the C:N ratio of feedstock or adding the appropriate amount of trace elements^47,48^.

When foaming occurs, a fast-responding defoaming agent can be added to efficiently suppress foam under the condition that the components do not inhibit the anaerobic digestion process or adversely affect the environment^49^. For example, Ca^2+^ and Mg^2+^ tend to combine with VFAs to form insoluble salts, and therefore decrease the surface active properties of VFAs^12^. Furthermore, the addition of Ca^2+^ and Mg^2+^ can increase the alkalinity in digesters and reduce inhibition on methanogenic bacteria due to acidification. In addition, reducing the OLR or ceasing feeding for a period of time can limit the production of surfactants (VFAs, NH_4_^+^, EPS, etc.) due to the hydrolysis and acidification of new substrate, and thereby ensure full degradation of accumulating surface active materials^8^. Moreover, reducing the OLR will decrease the substrate available to microbes, which will reduce the propagation of filamentous bacteria and biosurfactant producing bacteria. Increasing the stirring rate will also disrupt the growth of filamentous bacteria such as *Actinomyces*. Finally, to eliminate or reduce the contribution of accumulated NH_4_^+^ to foaming, ammonia stripping methods can be considered^48^.

## Materials and Methods

### Substrates and inoculum

Reactor substrates consisted of FW collected from a student dining hall at Chongqing University. Impurities such as bones, plastic, and paper were removed manually before the FW was ground into homogenized slurry. The FW slurry was stored at −18 °C and thawed at 4 °C for 1–2 days prior to use. The pH of the FW was 6.31 ± 0.21, total solids (TS) 28.20 ± 3.41%, volatile solids (VS) 26.61 ± 3.25%, VS to TS ratio (VS/TS) 93.61 ± 1.54%, and carbon content to nitrogen ratio (C/N) 14.73 ± 0.34.

The inoculum of RA consisted of sludge obtained from a rural household biogas pool operated at ambient temperature. The sludge was passed through a 10-mesh sieve to remove large inorganic particles, after which it was crushed and evenly mixed. To remove residual organic substrate, the sludge was incubated at 37 °C ± 1 °C for two weeks. The pH was 7.35 ± 0.13, TS 8.28 ± 0.63%, VS 5.54 ± 0.38%, VS/TS 67.05 ± 2.62%, and C/N 10.06 ± 0.43. The sludge for RB and RC inoculate was collected from the same biogas pool as that for RA and received the same pretreatments. The sludge was further centrifuged to meet the TS requirements for dry anaerobic digestion (20–40%)^50^. The pH of inoculum for RB was 7.54 ± 0.11, TS 23.53 ± 0.27%, VS 11.71 ± 0.13%, VS/TS 49.78 ± 0.14%, and C/N 8.06 ± 0.02. The sludge was acclimated for a period of time and then inoculated into RC. The pH was 7.54 ± 0.13, TS 26.15 ± 0.60%, VS 15.95 ± 0.56%, VS/TS 60.97 ± 0.73%, and C/N 10.02 ± 0.15.

### Experimental setup and operation

Three FW digesters were conducted semi-continuously under mesophilic temperature (37 °C ± 1 °C). RA was a CSTR with a total volume of 50 L and working volume of 30 L. Reactor contents were mixed intermittently at 90 rpm (3 h on/off) with an automatic motorized stirrer. Temperature in the reactor was maintained by a vessel jacket connected to a circulating hot water source. The initial OLR was 3.0 kgVS·m^−3^·d^−1^. Temperature fluctuations and stirring interruptions occurred on days 71, 72, and 75, and little foam appeared on the reactor surface. To enhance foaming, the OLR was increased to 4 gVS·L^−1^·d^−1^ on day 83. Intense foam appeared on day 87 and lasted for 43 days.

RB was a PFR with a total volume of 30 L and working volume of 18 L. The temperature control mode was the same as that for RA. A reflux ratio of 5:1 was used. The initial OLR was 3.0 kgVS·m^−3^·d^−1^, and OLR was increased following a specific gradient to 4.1, 5.5, 6.8, and 8.5 kgVS·m^−3^·d^−1^ after 1, 1, 2, and 3 hydraulic retention times (HRTs). The HRT for each stage was 45, 35, 25, 20, and 15 days, respectively. The air outlet clogged because of foaming in RB, and the internal pressure continuously increased, causing the reactor cover to crack on day 214.

RC was also a CSTR with the same volume and temperature control mode as RA. However a different mixing mode was used in RC. Reactor contents were mixed intermittently at 45 rpm (5 min on and 55 min off) with an automatic motorized stirrer. RC was operated with an OLR of 3.0 kgVS·m^−3^·d^−1^ until foam formation.

### Determination of physicochemical properties

Sludge (80 ml) was sampled daily before feeding for the physicochemical analysis. The pH, biogas production, and biogas composition for RA were monitored in-line. For RB and RC, pH was measured using a pH meter every day before feeding. Biogas production was measured using a wet gas flow meter, and biogas composition was determined every 3 days using a BIOGAS 5000 Portable Biogas Analyser (Geotech, UK). TS, VS, TA, TAN, VFAs, and C/N were analyzed according to methods described in our previous report^14^. Each indicator was determined in triplicate. VS reduction (VS_r_) and FAN concentration were calculated according to the method reported in our previous study^14^. Gas volume was corrected to standard temperature and pressure (0 °C and 101.325 kPa). Specific biogas production (SBP) and specific methane production (SMP) represent the volume of biogas or methane produced by adding 1 kg of organic matter based on VS content.

### Microbial community analyses

Microbial communities before and after foaming were investigated using Illumina MiSeq high-throughput sequencing. Three 10-mL sludge samples were collected in sterile 15-mL centrifuge tubes (LabServe, Ireland) from each of the reactors before and after foaming. The exact sampling times are shown in Fig. 1 and Supplementary Table S5. The sludge samples were stored at −80 °C immediately after collection until DNA extraction.

Total DNA was extracted from 0.3 g sludge with the E.Z.N.A.® Soil DNA Kit (Omega, USA) according to the manufacturer’s instructions. Extracted DNA from the same reactor and stage was mixed together and purified using the AxyPrep DNA gel Recovery Kit (AXYGEN, USA). The final DNA concentrations were determined by NanoDrop 2000 UV-vis spectrophotometer (Thermo Scientific, USA), and DNA quality was checked by 1% agarose gel electrophoresis. Primers for bacteria were 338 F (5′-ACTCC TACGG GAGGC AGCAG-3′) and 806R (5′-GGACT ACCAG GGTAT CTAAT-3′)^51,52^, and those for archaea were Arch344F (5′-ACGGG GYGCA GCAGG CGCGA-3′) and Arch915R (5′-GTGCT CCCCC GCCAA TTCCT-3′)^17,53^. All PCR reactions were performed in triplicate 20 μL mixtures containing 4 μL of 5 × FastPfu Buffer, 2 μL of 2.5 mM dNTPs, 0.8 μL of each primer (5 μM), 0.4 μL of FastPfu Polymerase (TransGen AP221-02, TransGen Biotech, China), 0.2 μL of BSA, 10 ng of template DNA, and ddH_2_O to a final volume of 20 μL. PCR amplification was performed in GeneAmp® 9700 (ABI, USA) under the following conditions: initial denaturation at 95 °C for 3 min, followed by 35 cycles of denaturation (95 °C for 30 s), annealing (55 °C for 30 s), extension (72 °C for 45 s), and a final extension at 72 °C for 10 min. The PCR products were purified by using AxyPrep DNA gel Recovery Kit (AXYGEN, USA) and quantified using QuantiFluor™-ST (Promega, USA) according to the manufacturer’s protocol.

A MiSeq library was prepared using the TruSeq™ DNA Sample Prep Kit (Illumina, USA) according to the manufacturer’s protocol. The main steps were as follows: (i) linking of ‘Y’ adapters; (ii) removal of adapter dimers with beads; (iii) PCR amplification to determine library concentrations; and (iv) generation of single-strand DNA fragments using sodium hydroxide. The sample libraries were pooled, and paired-end sequencing (2 × 300 bp) was conducted with an Illumina MiSeq platform (Illumina, USA) according to the standard protocols (Majorbio Bio-Pharm Technology Co. Ltd., Shanghai, China). The raw reads were deposited into the NCBI Sequence Read Archive (SRA) database with accession No. SRP091801.

Raw fastq files were demultiplexed and quality-filtered using QIIME software (version 1.17 http://qiime.org/scripts/assign_taxonomy.html) with the following criteria: (i) the reads were truncated at any site receiving an average quality score of <20 over a 50-bp sliding window; (ii) primers were matched allowing a maximum of two mismatched nucleotides, and reads containing ambiguous bases were removed; and (iii) sequences with overlaps longer than 10 bp were merged according to the overlapping sequence. Reads that could not be assembled were discarded. Operational Taxonomic Units (OTUs) were clustered with a 97% similarity cutoff using UPARSE (version 7.1 http://drive5.com/uparse/) and chimeric sequences were identified and removed using UCHIME. Shannon and Simpson diversity indices as well as species richness estimators, ACE and CHAO1, were obtained using the MOTHUR program (version 1.30.1 http://www.mothur.org/wiki/Schloss_SOP#Alpha_diversity). Sequence reads were normalized before estimation of diversity indices (i.e., normalized in mothur to the sample with the lowest number of sequences) (Supplementary Table S1). The phylogenetic affiliation of each 16S rRNA gene sequence was analyzed with the RDP Classifier (version 2.2 http://sourceforge.net/projects/rdp-classifier/) against the Silva (vesion SSU119 http://www.arb-silva.de) 16S rRNA database using a confidence threshold of 70%.

### Statistical analysis

Descriptive statistics, including mean and standard deviation (s.d.), were carried out for all physicochemical data using PASW Statistics. An unpaired two-tailed t-test was applied to compare differences between physicochemical indicators for different operation stages with an alpha level of 0.05. PCoA was performed on the weighted UniFrac phylogenetic distance matrices using R statistical software (http://www.r-project.org/) to visualize differences in microbial community compositions. The two-sided Chi-square test with DP, asymptotic with CC CI method, and fdr multiple test correction were conducted to identify significant differences between bacterial relative abundances in samples before and after foaming using STAMP software^54^.

### Data Availability

The original sequencing data are available at the NCBI SRA database with accession No. SRP091801. Other datasets supporting the conclusions of this article are included within the article and its Supplementary Informatica files.

## Electronic supplementary material

Supplementary Information
