# Supplementary material for: Investigation of foaming causes in three mesophilic food waste digesters: reactor performance and microbial analysis
Source: Sci Rep. 2017 Oct 20;7:13701. doi: 10.1038/s41598-017-14258-3 (PMC5651842; doi:10.1038/s41598-017-14258-3)
Supplement: Supplementary file 1 — Supplementary Information [file 41598_2017_14258_MOESM1_ESM.pdf]

Investigation of foaming causes in three mesophilic food waste digesters: reactor performance and microbial analysis

Qin He, Lei Li, Xiaofei Zhao, Li Qu, Di Wu, and Xuya Peng*

Key Laboratory of Three Gorges Reservoir Region’s Eco-Environment, Ministry of Education, Chongqing University, Chongqing 400045, PR China

*Corresponding author. E-mail: xuyapengcqu@126.com

Qin He, E-mail: hq88221@126.com

Lei Li, E-mail: lileicqu@126.com

Xiaofei Zhao, E-mail: 553289421@qq.com

Li Qu, E-mail: quliscu@163.com

Di Wu, E-mail: 389878075 @qq.com

Sample ID[a]	Sequences no	OTU	Good's coverage ,%	ACE	CHAO1	Shannon	Simpson
**Bacteria**							
RA-1	21569	280	99.77	320	313	3.82	0.042
RA-2	21569	253	99.73	298	315	2.99	0.126
RB-1	21569	348	99.65	425	423	3.91	0.053
RB-2	21569	307	99.67	367	394	3.49	0.086
RC-1	21569	297	99.73	343	349	3.53	0.067
RC-2	21569	353	99.74	343	338	3.44	0.073
**Archaea**							
RA-1	15629	35	99.97	39	37	1.08	0.565
RA-2	15629	80	99.94	87	85	1.73	0.294
RB-1	15629	67	99.94	73	72	1.70	0.366
RB-2	15629	40	99.92	69	59	0.85	0.688
RC-1	15629	88	99.93	95	95	1.55	0.499
RC-2	15629	78	99.94	84	84	1.24	0.595

Table S1. Statistics analysis of microbial sequence in samples retrieved from different reactors.

[a] RA-1 and RA-2 represented samples from RA before and after foaming, respectively; RB-1 and RB-2 represented samples from RB before and after foaming, respectively; RC-1 and RC-2 represented samples from RC before and after foaming, respectively. OUT: Operational taxonomic units.

Genera name	RA-2 (%)	RA-1 (%)	P value (corrected)	Difference (%)
Petrimonas	29.340	3.719	0	25.620
Aminobacterium	12.580	0.469	0	12.110
Actinomyces	12.020	1.332	0	10.690
Proteiniphilum	10.350	1.378	0	8.976
Gelria	5.512	1.174	1.253E-180	4.338
Family_XI_uncultured	3.384	1.221	5.303E-66	2.163
060F05-B-SD-P93	2.677	0.014	6.055E-164	2.663
Erysipelotrichaceae_UCG-004	1.815	0.215	1.38E-79	1.600
Syntrophomonadaceae_uncultured	1.727	7.080	1.235E-218	-5.353
Syntrophomonas	1.653	9.192	0	-7.539
Ruminiclostridium	1.017	0.014	3.959E-61	1.003
vadinBC27_wastewater-sludge_group	0.980	6.837	5.54E-294	-5.857
Gallicola	0.855	0.154	1.36E-31	0.701
Sedimentibacter	0.764	7.424	0	-6.659
Fastidiosipila	0.727	2.173	3.638E-48	-1.445
Ruminococcaceae_NK4A214_group	0.721	0.018	2.464E-42	0.703
Defluviitalea	0.690	1.045	5.1E-6	-0.355
Candidate_division_WS6_norank	0.680	9.589	0.000E+00	-8.909
OPB54_norank	0.610	0.115	2.543E-22	0.495
Clostridium_sensu_stricto_1	0.579	1.450	1.473E-25	-0.871
Bacteroidetes_VC2.1_Bac22_norank	0.502	6.640	0.000E+00	-6.138
Ruminococcaceae_unclassified	0.488	0.079	1.267E-19	0.410
Anaerolineaceae_uncultured	0.468	2.531	8.813E-94	-2.063
Candidatus_Caldatribacterium	0.357	0.186	1.233E-04	0.171
Synergistaceae_unclassified	0.350	0.036	4.61E-17	0.314
[Eubacterium]_coprostanoligenes_group	0.323	1.550	1.929E-53	-1.227
Acholeplasma	0.310	0.515	1.454E-04	-0.206
Terrisporobacter	0.296	0.694	1.157E-11	-0.398
Bacteroidetes_vadinHA17_norank	0.276	0.755	1.015E-15	-0.479
Christensenellaceae_R-7_group	0.216	1.818	1.718E-83	-1.603
Clostridiales_unclassified	0.205	0.093	7.674E-04	0.112
Turicibacter	0.202	0.440	5.534E-7	-0.238
Caldicoprobacter	0.175	0.018	4.416E-9	0.157
Rikenellaceae_RC9_gut_group	0.152	1.611	6.643E-80	-1.459
WCHB1-69_norank	0.138	0.319	7.546E-6	-0.181
Clostridiales_vadinBB60_group_norank	0.135	0.047	8.022E-04	0.088
Thermovirga	0.135	0.304	1.838E-5	-0.170
Bacteroides	0.125	0.444	7.031E-13	-0.319
Streptococcus	0.121	6.214	0	-6.093
Advenella	0.115	0.021	4.52E-5	0.093

Table S2. **Relative abundance and statistical analysis of the difference between the relative abundances of bacterial communities in RA before and after foaming**. Only bacterial genera that significantly changed their abundance after foaming and with relative abundance higher than 0.1% in foaming samples are represented.

Genera name	RB-2 (%)	RB-1 (%)	P value (corrected)	Difference (%)
Actinomyces	24.540	1.711	0	22.830
Candidate_division_WS6_norank	15.130	18.700	2.708E-30	-3.572
Fastidiosipila	10.580	7.894	1.425E-28	2.688
vadinBC27_wastewater-sludge_group	5.383	11.280	2.262E-146	-5.900
Clostridiales_unclassified	4.431	1.022	2.558E-135	3.409
Petrimonas	3.862	2.211	2.406E-30	1.651
Family_XI_uncultured	3.505	4.817	3.245E-15	-1.312
Aminobacterium	3.146	0.395	1.723E-133	2.751
Anaerolineaceae_uncultured	2.623	4.592	5.095E-37	-1.969
Bacteroides	2.457	1.580	1.257E-13	0.877
Christensenellaceae_R-7_group	1.904	3.867	2.201E-45	-1.963
Sedimentibacter	1.488	2.139	5.326E-9	-0.651
Anaerosalibacter	1.285	0.384	1.338E-31	0.901
Synergistaceae_uncultured	1.245	3.255	2.799E-60	-2.010
Ruminococcaceae_NK4A214_group	1.188	0.671	1.646E-10	0.518
Georgenia	1.015	1.765	1.495E-14	-0.750
Acholeplasma	0.899	0.663	1.627E-03	0.236
Ruminococcaceae_unclassified	0.762	0.268	4.093E-16	0.494
Tissierella	0.636	0.413	2.922E-04	0.223
Defluviitalea	0.613	0.007	4.362E-37	0.605
Treponema_2	0.606	2.338	4.964E-68	-1.732
Marinilabiaceae_norank	0.603	0.754	3.031E-02	-0.151
Mobilitalea	0.569	0.239	9.408E-10	0.330
Guggenheimella	0.556	0.388	4.105E-03	0.168
Streptococcus	0.536	5.277	2.06E-259	-4.741
Family_XI_unclassified	0.499	0.000	1.796E-31	0.499
Proteiniphilum	0.489	0.286	1.289E-04	0.203
Clostridium_sensu_stricto_1	0.353	0.518	3.063E-03	-0.165
Syntrophaceticus	0.350	0.004	9.335E-22	0.346
Synergistaceae_unclassified	0.330	1.754	4.766E-65	-1.425
Atribacteria_norank	0.313	0.732	3.061E-12	-0.419
Thermovirga	0.303	2.853	3.714E-137	-2.550
Caldicoprobacter	0.273	0.065	3.974E-9	0.208
Corynebacterium	0.246	0.159	3.373E-02	0.087
Pseudomonas	0.236	0.417	1.825E-04	-0.181
Desulfobulbus	0.206	0.094	8.446E-04	0.112
Blvii28_wastewater-sludge_group	0.186	0.268	4.773E-02	-0.082
Tepidimicrobium	0.176	0.033	2.842E-7	0.144
Peptococcaceae_uncultured	0.160	0.308	2.954E-04	-0.148
Chloroflexi_uncultured	0.160	0.319	1.191E-04	-0.159
[Eubacterium]_coprostanoligenes_group	0.160	0.479	1.257E-11	-0.319
Ruminiclostridium	0.143	0.025	3.234E-6	0.118
Cryptanaerobacter	0.107	0.207	3.178E-03	-0.100

Table S3. Relative abundance and statistical analysis of the difference between the relative abundances of bacterial communities in RB before and after foaming. Only bacterial genera that significantly changed their abundance after foaming and with relative abundance higher than 0.1% in foaming samples are represented.

Genera name	RC-2 (%)	RC-1 (%)	P value (corrected)	Difference (%)
Fastidiosipila	26.800	28.600	6.994E-6	-1.805
Actinomyces	16.200	5.262	9.502E-318	10.940
Proteiniphilum	13.860	9.615	8.306E-48	4.248
Family_XI_uncultured	4.880	5.952	1.175E-7	-1.072
vadinBC27_wastewater-sludge_group	4.768	9.717	2.503E-105	-4.949
Ruminococcaceae_unclassified	3.737	1.929	2.196E-32	1.809
Synergistaceae_unclassified	2.901	1.409	8.348E-29	1.492
Christensenellaceae_R-7_group	2.372	3.783	2.523E-20	-1.411
Ruminococcaceae_NK4A214_group	2.345	1.080	5.462E-26	1.264
[Eubacterium]_coprostanoligenes_group	2.266	8.734	2.915E-239	-6.468
Aminobacterium	2.072	0.899	1.319E-25	1.172
Petrimonas	1.990	1.423	1.736E-6	0.567
Bacteroides	1.375	0.881	3.503E-7	0.495
Lachnospiraceae_unclassified	1.287	3.282	2.82E-53	-1.996
Ruminococcaceae_UCG-014	1.256	4.066	4.347E-91	-2.810
Syntrophomonas	1.194	2.040	3.355E-14	-0.845
Mobilitalea	1.089	0.885	2.581E-02	0.203
Synergistaceae_uncultured	0.382	0.255	1.632E-02	0.127
Cryptanaerobacter	0.205	0.473	1.749E-7	-0.268
Sphaerochaeta	0.195	0.093	5.049E-03	0.102
Marinilabiaceae_norank	0.164	0.278	7.553E-03	-0.114
Clostridiaceae_unclassified	0.164	0.000	6.584E-9	0.164
Ruminococcus	0.157	0.014	5.83E-7	0.143
Mariniphaga	0.126	0.218	1.619E-02	-0.092
Corynebacterium	0.123	0.158	1.313E-02	-0.060
Lactobacillus	0.116	0.009	1.659E-5	0.107

Table S4. Relative abundance and statistical analysis of the difference between the relative abundances of bacterial communities in RC before and after foaming. Only bacterial genera that significantly changed their abundance after foaming and with relative abundance higher than 0.1% in foaming samples are represented.

Sample ID	Sampling time	Raw reads	Final reads	Reads lost during quality control	Final reads length, bp
**Bacteria**					
RA-1	Day 70	38358	27926	10432	438.98
RA-2	Day 100	45472	29728	15744	437.82
RB-1	Day 190	42963	27589	15374	433.82
RB-2	Day 214	45673	30036	15637	434.24
RC-1	Day 202	32735	21569	11166	432.44
RC-2	Day 232	46257	29286	16971	433.88
**Archaea**					
RA-1	Day 70	75402	34017	41385	270.08
RA-2	Day 100	68458	30255	38203	270.14
RB-1	Day 190	42231	15629	26602	267.88
RB-2	Day 214	38910	19136	19774	271.84
RC-1	Day 202	41410	21080	20330	267.39
RC-2	Day 232	58234	24993	33241	271.87

Table S5. Sequencing data in samples retrieved from different reactors.
